# Optimising a urinary extraction method for non-targeted GC–MS metabolomics

**DOI:** 10.1038/s41598-023-44690-7

**Published:** 2023-10-16

**Authors:** Cara Olivier, Bianca Allen, Laneke Luies

**Affiliations:** Human Metabolomics, North-West University, Potchefstroom Campus, Private Bag X6001, Box 269, Potchefstroom, 2520 NW South Africa

**Keywords:** Metabolomics, Gas chromatography, Liquid-liquid extraction

## Abstract

Urine is ideal for non-targeted metabolomics, providing valuable insights into normal and pathological cellular processes. Optimal extraction is critical since non-targeted metabolomics aims to analyse various compound classes. Here, we optimised a low-volume urine preparation procedure for non-targeted GC–MS. Five extraction methods (four organic acid [OA] extraction variations and a “direct analysis” [DA] approach) were assessed based on repeatability, metabolome coverage, and metabolite recovery. The DA method exhibited superior repeatability, and achieved the highest metabolome coverage, detecting 91 unique metabolites from multiple compound classes comparatively. Conversely, OA methods may not be suitable for all non-targeted metabolomics applications due to their bias toward a specific compound class. In accordance, the OA methods demonstrated limitations, with lower compound recovery and a higher percentage of undetected compounds. The DA method was further improved by incorporating an additional drying step between two-step derivatization but did not benefit from urease sample pre-treatment. Overall, this study establishes an improved low-volume urine preparation approach for future non-targeted urine metabolomics applications using GC–MS. Our findings contribute to advancing the field of metabolomics and enable efficient, comprehensive analysis of urinary metabolites, which could facilitate more accurate disease diagnosis or biomarker discovery.

## Introduction

Metabolomics refers to the quantitative measurement of dynamic metabolic changes in a system responding to genetic modifications or physiological stimuli, either external (e.g., drugs) or internal (e.g., nutrients)^1^. It involves the analysis of the metabolome, which can be defined as the complete set of small molecules (metabolites) present in a biological sample. Multi-level profiling of metabolites can be conducted in an unbiased manner through the comparison of sets of biological samples^2^.

Gas chromatography mass spectrometry (GC–MS) is considered the “gold standard” for metabolomics studies^3^ due to its many advantages, including its suitability for both volatile and non-volatile compound analyses (after derivatization), affordability in terms of low running costs, high sensitivity with excellent chromatographic and mass resolution, good dynamic range, and compound identification using mass spectral library matching (of which extensive commercial libraries are available). Furthermore, GC–MS provides additional and orthogonal data (such as retention time), reproducible chromatographic separations (making results comparable between different laboratories and analysts), and the ability to distinguish stereoisomers. GC–MS also has shorter run times with lower bleed^4^.

Although GC–MS has some disadvantages, such as the co-elution of compounds and slower scan rates, most of these can be compensated for, for example, with two-dimensional coupling (GCxGC) or by coupling the system to time-of-flight mass spectrometry (TOFMS), respectively^5^. These approaches broaden the application of GC–MS substantially^6^. However, since GC–MS is limited to volatile, thermally stable compounds—and most natural occurring compounds in a biological matrix are non-volatile and unstable at high temperatures—samples need to be derivatized prior to analysis^7^. Derivatization refers to the process of chemically modifying a compound, exploiting its polarity, and boiling point, including the interaction with a GC’s capillary column to achieve compound separation. Hence, most methods require two-step derivatization, employing oximation followed by silylation^8^, although the latter is often used as a one-step derivatization approach. These derivatization methods will be further explored in this publication.

Urine has proven to be an excellent sample matrix for non-targeted metabolomics studies because it reflects both normal and pathological cellular processes, providing a holistic assessment of metabolic profiles. Urine is a particularly valuable sample matrix due to its ease of collection, straightforward handling and processing in the laboratory, and the reduced risk of nosocomial transmission to healthcare and laboratory personnel. These advantages make urine a promising alternative biological sample, especially given the challenges in the collection and yield of other biological samples in certain diseases^9^. Early therapy intervention and improved treatment effectiveness depend on the insights derived from urine metabolomics. Therefore, urine metabolomics can be employed to rule out potential metabolic defects, assess nutritional issues, and detect signs of infections or overgrowth (including pathogen or flora). To maximise the utility of urinary metabolome data for disease characterisation and treatment strategies, effective sample preparation, coupled with selective extractions tailored to the chosen analytical technique, becomes critically important^10^.

For urine GC–MS analyses, organic acid (OA) metabolite extraction remains a popular option. The principle of an OA extraction is typically based on a two-step liquid–liquid extraction (LLE; also known as partitioning)^11^ which involves the use of two immiscible solvents^12^. However, LLE is better suited for targeted investigations in clinical settings, such as diagnosing organic acidurias^4^. Optimal extraction is critical since non-targeted metabolomics aims to analyse various compound classes at varying concentrations. To this end, recent advances in technology have led to the development of more efficient and cost-effective methods for compound extraction, such as the direct analysis (DA) method, for non-targeted metabolomics.

The DA method involves deproteinisation, sample concentration, and derivatization^13^. Although straightforward, various studies suggest an additional drying step between two-step derivatization^14,15^ to improve reproducibility (since residual water and other contaminants can be removed), reduce matrix effects (by removing water and other impurities that can interfere with ionisation and compound detection), and increase sensitivity (since background noise is reduced while the signal-to-noise ratio is increased).

One significant challenge in non-targeted metabolomics using urine samples, is the presence of urea, which can obscure other metabolites in the sample with the same retention time and/or interfere with the derivatization process, leading to incomplete chemical transformation and the formation of urea-derived artifacts^7,8^. To address this issue, various studies suggest using the enzyme urease to remove excess urea from urine samples prior to GC–MS analysis^16,17^. This also has the advantage of avoiding column overloading, peak distortions, lower chances of co-eluting peaks, and lower coefficient of variation (CV) values compared to urease-non-treated samples^16,18^. However, the use of urease pre-treatment has its disadvantages, including the occurrence of unwanted chemical transformations and secondary enzymatic reactions that can alter the metabolic profile^19,20^, and the inability to detect several compounds in urease-treated samples compared to non-treated samples^16^. As a result, the use of urease pre-treatment in non-targeted metabolomics remains a subject of debate in the scientific community.

In this investigation, we aimed to optimise a low-volume urine preparation procedure for non-targeted GC–MS analyses, covering various compound classes. In Part A, five methods were evaluated based on repeatability, metabolome coverage, and metabolite recovery. An OA extraction method (Method 1) was compared with modified versions, namely the use of an additional extraction solvent (Method 2), two-step derivatization (Method 3), and a combination of Methods 2 and 3 (Method 4). A DA method (Method 5) was also tested. In Part B, the superior method was further optimised by investigating if an additional drying step between two-step derivatization (Method 6) and urease sample pre-treatment (Method 7) is indeed beneficial for disease characterisation studies.

## Methods

### Chemicals

The following reagents were acquired from Sigma Aldrich (St. Louis, Missouri, USA) and Merck (Darmstadt, Germany): 3-phenylbutyric acid, methoxyamine hydrochloride (MOX-HCl), anhydrous sodium sulphate (Na_2_SO_4_), urease, glucose, 4-aminobutyric acid (GABA), L-alanine, L-leucine, L-phenylalanine, L-tryptophan, L-tyrosine, ascorbic acid, citric acid, succinic acid, palmitic acid, arabinose, ribose, N,O-bis(trimethylsilyl)trifluoroacetamide (BSTFA) with 1% trimethylsilyl chloride (TMCS), and pyridine. Other chemicals used were acetonitrile, ethyl acetate, hydrogen chloride and diethyl ether from Burdick and Jackson brand (Honeywell International Inc., Muskegon, USA).

### Standards

A spiking mixture/solution (50 ppm) was prepared prior to the experiments, using known amounts of compounds from different compound classes. This mixture was used to determine the percentage recovery of the known compounds introduced to the samples. Three different compound classes were included: amino acids (GABA, L-alanine, L-leucine, L-phenylalanine, L-tryptophan, L-tyrosine), organic acids (ascorbic acid, citric acid, succinic acid), and a fatty acid (palmitic acid).

An internal standard (IS; 50 ppm) solution containing 3-phenylbutyric acid was also prepared separately.

### Sample preparation and ethical approval

The investigation was done according to the Declaration of Helsinki and International Conference of Harmonization guidelines. Ethical approval was obtained from the Ethics Committee of the North-West University for a larger study (NWU-00355-20-A1), as well as for this sub-study (NWU-00355-20-A1-03). All recruited participants gave written informed consent.

A single quality control (QC) sample, compiled from ethically approved, previously collected healthy urine samples (n = 32) from which multiple smaller (100 μL) aliquots were made, was used in this experiment. Before an aliquot was transferred, the material was thoroughly mixed by vortex. The sample aliquots were thawed at room temperature, and to eliminate any crystals and make sure that any precipitated material has dissolved, the urine was centrifuged at 15 700×g for 5 min at room temperature.

### Metabolite extraction

**Figure 1:**
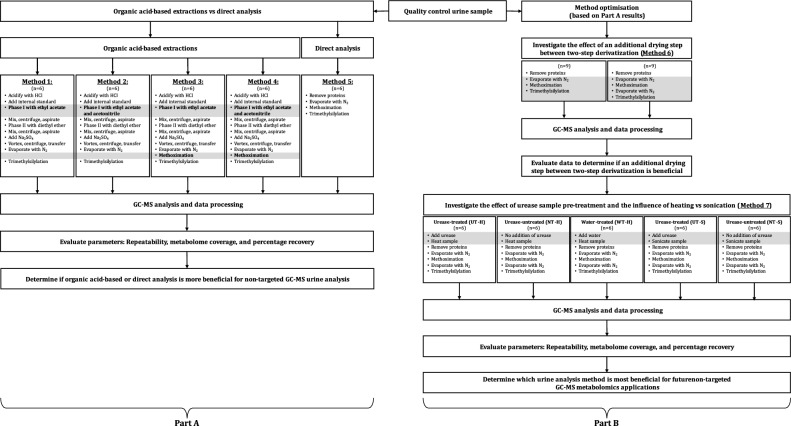
Summary of the experimental design. This study comprises a Part A (to evaluate five extraction methods) and Part B (to optimise a urine preparation procedure for non-targeted GC–MS analyses). The variations between the methods are indicated in grey sections. For Methods 1–5, six QC aliquots were used each, while Method 6 required 18 aliquots and Methods 7 needed 30 aliquots.

An illustration of the experimental design is available in Fig. 1. For Part A of this investigation, five methods were evaluated based on repeatability, metabolome coverage, and metabolite recovery. An OA extraction method (previously optimised for a reduced urine volume; Method 1) was compared with modified versions of this method, namely the use of acetonitrile as an additional extraction solvent (Method 2), two-step derivatization to include both oximation and silylation (Method 3), as well as a combination of Methods 2 and 3 (Method 4). These methods were also compared to a DA method (Method 5).

In Part B, the superior method was further optimised by investigating the effect(s) of an additional drying step between two-step derivatization (Method 6). Hereafter, urease sample pre-treatment was evaluated (Method 7) by comparing urease-treated samples to both urease-non-treated and water-treated samples. Furthermore, the effect of the heating process that urease pre-treatment requires was investigated by comparing the urease-treated to urease-non-treated samples subjected to both heating and sonication.

Methods 1–5 was conducted as a single experiment, for which six QC aliquots were used each (n = 30), while Method 6 was conducted in a second experiment, requiring 18 aliquots, and Method 7 as a third experiment, which needed 30 aliquots.

#### *Method 1 (traditional OA extraction)*^21^

Six drops of 5N HCl were added to 100 μL urine samples filled up to 500 μL with a 9% saline solution (n = 6), followed by the addition of IS (100 μL, final concentration of 50 ppm). Hereafter, half of the samples (n = 3) were spiked (referred to as the “pre-spike samples”), and ethylacetate (6 mL) was added to all samples. The samples were mixed (30 min) and centrifuged (845xg for 5 min at 4 °C), and the organic phase was collected. Diethylether (3 mL) was added to the remaining aqueous phase, and the samples were again mixed, centrifuged, and the organic phase collected and combined with the previously collected phase. Approximately 3 g Na_2_SO_4_ was added to each sample's organic phases, followed by vortex and centrifugation. The organic phase was transferred to a new tube and the remaining half of the samples were spiked (n = 3; referred to as the “post-spike samples”). All samples were completely dried under a light stream of nitrogen gas at 37 °C. Silylation was performed with 50 μL BSTFA with 1% TMCS and 50 μL pyridine, with incubation at 60 °C for 60 min. Hereafter, samples were allowed to cool to room temperature and transferred to a GC sample vial with an insert and again capped.

#### *Method 2 (extraction solvent evaluation)*^22^

This method was identical to that of Method 1, except for the addition of 1.5 mL ice-cold acetonitrile with ethylacetate: Six drops of 5N HCl were added to 100 μL urine filled up to 500 μL with a 9% saline solution (n = 6). Hereafter, the IS (100 μL, final concentration of 50 ppm) was added and half of the samples (n = 3) were spiked (“pre-spike samples”). Ethylacetate (6 mL) was added to all samples, followed by ice-cold acetonitrile (1.5 mL), whereafter samples were mixed (30 min) and centrifuged (845xg for 5 min at 4 °C). After the organic phase was collected, diethylether (3 mL) was added to the remaining aqueous phase, and the samples were again mixed, centrifuged, and the organic phase collected and combined with the previously collected phase. Approximately 3 g Na_2_SO_4_ was added to the organic phases of each sample, followed by vortex and centrifugation. The organic phases were then transferred to a new tube and the remaining half of the samples were spiked (n = 3; “post-spike samples”). All samples were evaporated to complete dryness under a light stream of nitrogen gas at 37 °C. Silylation was performed with BSTFA with 1% TMCS (50 μL) and pyridine (50 μL), with incubation at 60 °C for 60 min. Hereafter, samples were allowed to cool to room temperature and transferred to a GC sample vial with an insert and again capped.

#### *Method 3 (one-step versus two-step derivatization)*^23^

This method was identical to that of Method 1, except for an addition methoximation step prior to silylation (a two-step derivatization): After the addition of six drops of 5N HCl and a 50 ppm IS (100 μL) to urine samples (n = 6; 100 μL filled up to 500 μL with a 9% saline solution), half of the samples (n = 3) were spiked (“pre-spike samples”). Hereafter, and ethylacetate (6 mL) was added to all samples, followed by mixing (30 min), centrifugation (845xg for 5 min at 4 °C), and organic phase collection. Diethylether (3 mL) was added to the remaining aqueous phase, and the samples were again mixed, centrifuged, and the second organic phase collected and combined with the first. Na_2_SO_4_ (approximately 3 g) was added to the collected organic phases, followed by vortex and centrifugation. Hereafter, the samples were transferred to a new tube and the remaining half were spiked (n = 3; “post-spike samples”). All samples were completely dried under a light stream of nitrogen gas at 37 °C prior to methoximation. Thus, 50 μL MOX-HCl dissolved in pyridine (20 mg/mL) was added to each sample, capped, and incubated at 50 °C for 90 min. Hereafter, the samples were left to cool to room temperature prior to silylation with 50 μL BSTFA with 1% TMCS (no additional pyridine was added), with incubation at 60 °C for 60 min. The samples were again allowed to cool to room temperature and transferred to a GC sample vial with an insert and re-capped.

#### Method 4 (combination of Methods 2 and 3)

This method was identical to that of Methods 2 and 3, combining the use of ice-cold acetonitrile with ethylacetate, and two-step derivatization: To each urine sample (n = 6; 100 μL urine filled up to 500uL with a 9% saline solution), six drops of 5N HCl and 100 μL of an IS (500 ppm) was added. Hereafter, half of the samples were spiked (n = 3; “pre-spike samples”). Ethylacetate (6 mL) and ice-cold acetonitrile (1.5 mL) was added to all sample, followed by mixing (30 min), centrifugation (845xg for 5 min at 4 °C), and organic phase collection. Diethylether (3 mL) was then added to the remaining aqueous phase, again followed by mixing, centrifugation, and the organic phase collection (which was combined with the previously collected phase). Na_2_SO_4_ (approximately 3 g) was added to the collected organic phases, and after vortex and centrifugation, the samples were transferred to a new tube. The remaining half of the samples were then spiked (n = 3; “post-spike samples”). All samples were dried with nitrogen gas at 37 °C. Once samples were completely dry, methoximation was performed using 50 μL MOX-HCl dissolved in pyridine (20 mg/mL). Samples were capped and incubated at 50 °C for 90 min. Hereafter, the samples were left to cool to room temperature prior to silylation with 50 μL BSTFA with 1% TMCS (no additional pyridine was added), with incubation at 60 °C for 60 min. The samples were again allowed to cool to room temperature, before transferring them to a GC sample vial with an insert, and re-capped.

#### *Method 5 (DA)*^13^

The IS (100 μL; final concentration of 50 ppm) was added to 100 μL urine. Half of the samples were spiked (n = 3; “pre-spike samples”), ice-cold acetonitrile (300 μL) was added, and samples were centrifuged at room temperature (15000xg for 5 min). The supernatant was collected and transferred to a GC–MS vial, whereafter the remaining half of the samples were spiked (n = 3; “post-spike samples”). Similar as before, the samples were dried completely under a light stream of nitrogen gas at 37 °C. The vials were allowed to cool to room temperature before applying two-step derivatization: Samples were methoxylated using 50 μL MOX-HCl dissolved in pyridine (20 mg/mL), capped, and incubated at 50 °C for 90 min. After cooling to room temperature, samples were silylated with 50 μL BSTFA with 1% TMCS (no additional pyridine was added), capped, and incubated at 60 °C for 60 min. Samples were transferred to a glass insert, placed into the original GC–MS vial, and capped.

#### *Method 6 (additional drying step)*^15^

For this part of the investigation, 18 QC aliquots were used, divided into two groups: Group 1 samples (n = 9) were subjected to Method 5 exactly as indicated above (including two-step derivatization). Group 2 samples (n = 9) were also subjected to Method 5, however, the derivatization process was adjusted by adding another drying step prior to silylation. Following methoximation as described above (50 μL MOX-HCl dissolved in pyridine [20 mg/mL]), samples were again dried under a light stream of nitrogen gas at 37 °C prior to silylation with 50 μL BSTFA with 1% TMCS (no additional pyridine was added). After cooling to room temperature, samples were transferred to a glass insert, placed into the original GC–MS vial, and capped.

#### Method 7 (urease pre-treatment)

QC aliquots (n = 30) were divided into five groups, namely Group 1 urease-treated samples subjected to heating (n = 6; UT-H); Group 2 urease-non-treated samples subjected to heating (n = 6; NT-H); Group 3 water-treated samples subjected to heating to serve as a blank sample for investigating the diluting effect urease pre-treatment has on samples (n = 6; WT-H); Group 4 urease-treated samples subjected to sonication instead of heating (n = 6; UT-S); Group 5 urease-non-treated samples subjected to sonication (n = 6; NT-S). The analysis of these samples was based on Method 6, however, following the addition of the IS, urease (100 μL of a 1 mg/mL solution; Groups 1 and 4) or water (100 μL; Group 3) was also added. The samples were then either heated (30 min at 37 °C; Groups 1–3)^16^ or sonicated (30 min at room temperature with 005 power; Groups 4 and 5)^18^ for adequate reaction to occur, before continuing with the addition of ice-cold acetonitrile whereafter the rest of the method proceeded as described previously (see “Method 6”).

### GC–MS analysis

Prior to sample analysis, a routine clean-up and maintenance check was performed (i.e., leak check, tune check, and mass calibration) and a new liner and septum was inserted to prevent any undesired reactions and surface adsorption phenomena.

Samples were analysed in two batches (Part A; Methods 1–5) and (Part B; Methods 6 and 7), in a randomised manner. The samples were placed into an auto-sampler tray (Gerstel MS Germany), combined with a Pegasus 4D GCxGC-TOFMS system (LECO Africa [Pty] Ltd, Johannesburg, South Africa) and fitted with an Agilent 7890 GC and TOFMS (LECO Africa). All samples (1 μl) were injected using a 1:10 split ratio with purified helium as a carrier gas, set at a constant flow of 1.4 mL/min. Chromatographic separation was achieved with a Restek Rxi-5MS primary capillary column (28.2 m; 250 μm diameter; 0.25 μm film thickness), and a Restek Rxi-17 secondary capillary column (1.3 m; 250 μm diameter; 0.25 μm film thickness). Throughout the entire chromatographic run, the front inlet temperature was maintained at 250 °C, the transfer line at 225 °C, and the ion source was kept constant at 200 °C. Cryomodulation was achieved with a hot pulse of nitrogen gas for 0.7 s, every 3 s. The primary oven programme (see Figure S1 in the supplementary information) started at 70 °C, which was held for 1 min. The temperature was then ramped up as follows: 5 °C/min to 100 °C, 10 °C/min to 160 °C, 13 °C/min to 230 °C, and finally 20 °C/min to 300 °C, which was held for 2 min. The secondary oven was programmed identical to that of the primary oven, except for a + 5 °C at each interval. The total run time of each sample was ≈24 min. Prior to MS data acquisition, a 480 s solvent delay was used, during which no mass spectra were captured. However, to accurately represent retention times, this interval was appended to the time axis of the GC column. Mass spectra were collected over a range of 50–800 m/z at an acquisition rate of 20 spectra per second. The filament bias was -70 eV and the detector voltage was optimised at an offset of 50 V.

### Data analysis

For each experimental dataset, Leco® ChromaTOF-GC Software (v4.72.0.0) was used for MS deconvolution (with a signal-to-noise ratio of 100 and a minimum of three apexing peaks), peak identification (with a 60% similarity match) and peak alignment. The raw data from the GCxGC-TOFMS was exported as a .csv file and converted to an Excel Workbook (.xlsx) format. The data was normalised based on the IS, followed by normalisation based on the creatinine value. Various parameters were assessed using Excel, including analytical repeatability, metabolome coverage, and metabolite recovery.

## Results and discussion

In Part A of this investigation, five methods were evaluated based on repeatability, metabolome coverage, and metabolite recovery. The goal here was to select the most appropriate method for non-targeted GC–MS application. Hereafter, the selected method was further explored for possible optimisation in Part B.

### Repeatability

**Figure 2:**
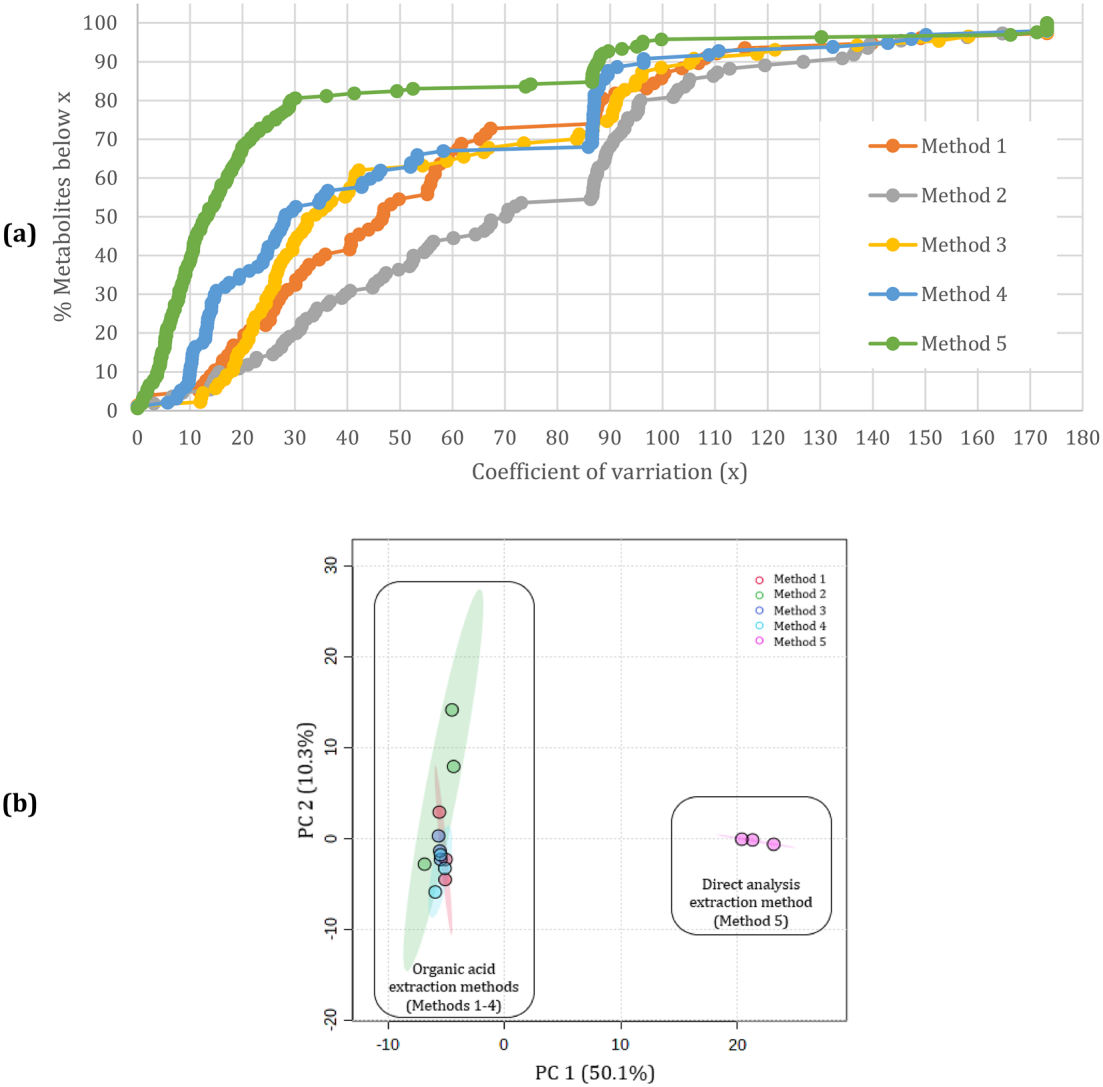
Evaluation of repeatability of the five extraction methods. (**a**) The percentage of metabolites below a coefficient of variation (CV) threshold < 50%, ranked as Method 5 (82.4%), Method 3 (62.1%), Method 4 (61.9%), Method 1 (54.6%), and Method 2 (36.4%). (**b**) Principal component analysis (PCA) scores plot which illustrates the clustering patterns among the samples based on their metabolite profiles/composition. The plot provides an overview of the overall similarity or dissimilarity among the methods’ samples without directly incorporating the CV values.

To determine the analytical repeatability for Methods 1–5, the first three samples of each method (i.e., the “pre-spike samples”) were used to calculate the CV values, expressed as percentage. When interpreting the CV values, the more overall compounds with a CV < 50%, the better the method^24^. Figure 2a illustrates the CV distribution for all detected compounds, for the five extraction methods. When considering the analytical repeatability, Method 5 had the best repeatability (82.4% of all detected compounds had a CV < 50%), followed by Method 3 (62.1%), Method 4 (61.9%), Method 1 (54.6%), and lastly Method 2 (36.4%).

For further confirmation of the above ranking, the web-based server MetaboAnalyst was used to statistically evaluate the data and compile a principal component analysis (PCA) scores plot (see Fig. 2b). This plot provides an overview of the overall similarity or dissimilarity among all five extraction methods without directly incorporating the CV values, but rather considering patterns and relationships among the samples of each method based on their measured variables. Indeed, the PCA analysis revealed distinct clustering patterns among the five methods. Specifically, the OA extraction methods (Methods 1–4) exhibited a tight clustering, suggesting similar metabolite profiles. The DA samples (Method 5) were positioned distinctly and significantly distant from the OA extraction methods. This observation suggests substantial dissimilarity in the metabolite profiles of Method 5 compared to the other methods, likely due to its unique extraction principle and variability in its metabolite composition. On a PCA, the approach with the least amount of variance will have the smallest 95% confidence region circle, which is where the samples cluster closest together. Thus, the bigger the 95% confidence region, the less repeatable the method. The large green ellipse, which represents Method 2 (i.e., the addition of acetonitrile), shows great variance between the samples. Although acetonitrile has a superior extraction efficiency, Majors (2013)^22^ also observed that its extracts were still somewhat "dirty", possibly contributing to the observed variation. Furthermore, the presence of water in acetonitrile is a potential factor in the variance between the extracted analytes, as it may impact how effectively different OAs are extracted, depending on how the OA is distributed between the aqueous and organic phases^25^. This may lead to the loss of compounds during extraction, making this method the least repeatable. Thus, it may be beneficial to add kosmotropic salts (e.g., MgSO_4_ or NaCl) to phase separate acetonitrile from a water/acetonitrile solution. The original in-house method samples (Method 1; red ellipse), has slight variation between the samples but less so compared to Method 2. Considering the additional compound stabilisation that oximation offers^23^, Method 3 (dark blue ellipse) and Method 4 (blue ellipse) have better confidence region circles than Methods 1 and 2, indicating less variability, which concurs with Fig. 2a. Similarly, Method 5 (pink ellipse), which also entails oximation prior to silylation, shows tight sample clustering. Thus, the method ranking based on the PCA scores plot corresponds with that of the CV-graph.

Based on Fig. 2, Method 5 yields more repeatable results compared to any of the OA extraction methods. This is possibly due to oximation for compound stabilisation, in conjunction with its easy preparation (i.e., reduced analytical steps involved) since the OA extraction methods are more labour-intensive and hence more prone to errors.

### Metabolome coverage

The metabolome coverage of each method was assessed using the normalised “pre-spike samples” only. The total number of compounds detected across all five extraction methods (n = 220) was determined, whereafter the number of overall compounds not detected in at least 50% of all samples for each extraction method was calculated as: $\mathrm{\%\, compounds\, undetected}=\frac{\mathrm{Number\, of\, compounds\, with\, zero\, average}}{220\,\mathrm{compounds\, overall}}\times 100$. The extraction methods were then ranked based on this calculated percentage, from the least number of undetected compounds to the most undetected compounds as follows: Method 5 (26.4% undetected); Method 2 (51.4% undetected); Method 4 (57.3% undetected); Method 3 (61.8% undetected); and Method 1 (66.4% undetected). Upon further investigation, we found that 91 metabolites were exclusively detected using Method 5, whereas 58 were specific to the four OA extraction methods. Of these 58, 13 were exclusive to Method 2, seven were exclusive to Method 4, one was exclusive to Method 3, and none was exclusive to Method 1 (see Table 1 for complete lists of these unique compounds). As can be seen, both metabolome coverage rankings (either considering the percentage of undetected compounds or the number of unique compounds) concurred.

**Table 1:** Unique compounds detected for each method, and those detected in only the organic acid extraction methods.

Method name and number of unique compounds	List of unique compounds			
Method 1 (n = 0)	None			
Method 2 (n = 13)	Analyte 003 Analyte 013 Analyte 121 Analyte 128	Analyte 135 Analyte 148 Analyte 150	Analyte 156 Analyte 157 Analyte 167	Analyte 171 Analyte 192 Analyte 308
Method 3 (n = 1)	Analyte 322			
Method 4 (n = 7)	2,3,4-Trihydroxy-butyraldehyde O-methyl-oxime	Analyte 036 Analyte 071	Analyte 209 Analyte 216	Analyte 325 Malonic acid
Method 5 (n = 91)	2-Amino-benzoic acid 3,4,5-Trihydroxy-pentanoic acid 3,4-Dihydroxy-butyric acid 3R,4S-Tetrahydrofuran-2,3,4-triol 4-Hydroxy-pyrrolidine-2-carboxylic acid 5-Hydroxymethyl-tetrahydro-furan-2,3,4-triol [1] 5-Hydroxymethyl-tetrahydro-furan-2,3,4-triol [2] 5-Oxo-pyrrolidine-2-carboxylic acid Analyte 004 Analyte 005 Analyte 023 Analyte 025 Analyte 029 Analyte 035 Analyte 038 Analyte 043 Analyte 046	Analyte 050 Analyte 057 Analyte 058 Analyte 061 Analyte 063 Analyte 064 Analyte 070 Analyte 072 Analyte 085 Analyte 086 Analyte 087 Analyte 090 Analyte 096 Analyte 099 Analyte 118 Analyte 125 Analyte 126 Analyte 131 Analyte 137 Analyte 145 Analyte 151 Analyte 177 Analyte 183 Analyte 186 Analyte 199	Analyte 210 Analyte 212 Analyte 218 Analyte 220 Analyte 225 Analyte 251 Analyte 253 Analyte 266 Analyte 276 Analyte 278 Analyte 280 Analyte 285 Analyte 287 Analyte 302 Analyte 305 Analyte 306 Analyte 311 Analyte 321 Analyte 323 Arabinofuranose Arabinose Ascorbic acid Aucubin Butane-2,3-diol Creatinine	Dehydroabietic acid Erythronic acid ç-lactone Galactose [1] Galactose [2] Glucose Glucuronic acid Glucuronic acid ç-lactone Glycerol Isocitric lactone meso-Erythritol Methyl galactoside Methylmalonic acid Myo-Inositol [1] Myo-Inositol [2] N-Acetyltyrosine N-benzoyl-glycine Oxalic acid Oxalic acid [1] Oxalic acid [2] Rhamnose Ribofuranose Tagatofuranose Threo-3-deoxy-pentonic acid Xylose
Compounds unique to the organic acid methods (Methods 1–4)* (n = 58)	2,3,4-Trihydroxy-butyraldehyde O-methyl-oxime 2,4-Dihydroxybutyric acid 2-Hydroxy-pentanedioic acid 5-Hydroxymethyl-tetrahydro-furan-2,3,4-triol [1] 5-Hydroxymethyl-tetrahydro-furan-2,3,4-triol [2] Analyte 003 Analyte 013 Analyte 014 Analyte 020 Analyte 022 Analyte 024	Analyte 027 Analyte 031 Analyte 036 Analyte 049 Analyte 052 Analyte 055 Analyte 059 Analyte 071 Analyte 121 Analyte 123 Analyte 128 Analyte 135 Analyte 143 Analyte 144 Analyte 148 Analyte 150	Analyte 156 Analyte 157 Analyte 167 Analyte 168 Analyte 170 Analyte 171 Analyte 173 Analyte 174 Analyte 175 Analyte 176 Analyte 192 Analyte 195 Analyte 205 Analyte 209 Analyte 216 Analyte 226	Analyte 239 Analyte 240 Analyte 255 Analyte 297 Analyte 301 Analyte 308 Analyte 314 Analyte 322 Analyte 325 Benzene-1,2,3-triol Citric acid *[miss ID]* Malonic acid diethyl ester Octadecanoic acid Propane-1,2,3-triol [1] Tetrahydro-pyran-2,3,4,5-tetraol (isomer 2)

*Some of these compounds were detected across multiple organic acid extraction methods, hence they are not unique to a specific organic acid method (Methods 1–4), however, they were not detected using the direct analysis method (Method 5).

### Metabolite recovery

A spiking mixture is a carefully prepared solution containing known concentrations of specific compounds (see Section "Standards"), and serves as a vital component in analytical chemistry, fulfilling various purposes. In this study, a spiking mixture was used to specifically assess the recovery rate of target analytes during sample preparation and analysis. This assessment involves adding a known amount of analyte into the natural test sample matrix. By comparing the amount of spike compounds added to the sample with the amount detected in the final analysis, researchers can calculate the recovery percentage, which indicates the efficiency of target analyte extraction and measurement. Thus, metabolite recovery was determined by comparing the concentrations of compounds in the “pre-spike” and “post-spike” samples, using only those compounds included in the spiking solution (see Section "Standards"). The percentage of recovery was calculated as:﻿ $\mathrm{\%\, recovery}=\frac{\mathrm{Pre-spike\, concentration}}{\mathrm{Post-spike\, concentration}}\times 100$(see Table 2).

**Table 2:** Metabolite recovery of the compounds in the spiking mixture (containing pure, known standard concentrations of different class compounds) indicated for each method. The compounds are grouped per class.

Spiking compound	Method 1 (original in-house)			Method 2 (acetonitrile addition)			Method 3 (two-step derivatization)			Method 4 (combined methods 2 & 3)			Method 5 (direct analysis)		
	Pre-spike average	Post-spike average	% Recovery	Pre-spike average	Post-spike average	% Recovery	Pre-spike average	Post-spike average	% Recovery	Pre-spike average	Post-spike average	% Recovery	Pre-spike average	Post-spike average	% Recovery
4-Aminobutyric acid (GABA)	0.00	0.74	0.00	0.00	0.95	0.00	0.00	0.84	0.00	0.00	0.23	0.00	0.11	0.35	30.46
L-Alanine	0.00	3.06	0.00	0.00	4.01	0.00	0.00	3.70	0.00	0.00	1.79	0.00	1.85	3.01	61.35
L-Leucine	0.00	1.54	0.00	0.00	0.18	0.00	0.00	1.47	0.00	0.00	0.23	0.00	0.45	0.99	45.66
L-Tryptophan	0.00	0.00	0.00	0.00	0.00	0.00	0.00	0.00	0.00	0.00	0.00	0.00	0.00	0.01	0.00
L-Tyrosine	0.00	0.28	0.00	0.00	0.24	0.00	0.00	0.37	0.00	0.00	0.17	0.00	0.00	0.03	0.00
L-Phenylalanine	0.00	0.53	0.00	0.00	0.65	0.39	0.09	0.78	11.94	0.03	0.45	6.15	0.21	0.24	86.55
Ascorbic acid	0.00	0.02	0.00	0.00	0.04	0.00	0.00	0.09	0.00	0.00	0.01	0.00	0.09	0.11	86.49
Citric acid	0.57	0.79	72.25	1.02	1.24	82.56	0.59	0.89	65.61	0.90	0.69	130.81	1.63	1.38	118.12
Succinic acid	0.11	0.13	82.33	0.13	0.15	85.62	0.11	0.14	80.08	0.10	0.12	79.05	0.16	0.21	78.77
Palmitic acid	0.14	0.21	67.57	0.38	0.21	181.00	0.17	0.34	50.67	0.12	0.18	67.16	0.09	0.13	69.28
Average recovery			**22.22**			**34.96**			**20.83**			**28.32**			**57.67**

Among the evaluated methods, Method 5 exhibited an average percentage recovery of 57.7%. Notably, citric acid displayed the highest recovery (118.1%), followed by phenylalanine (86.6%), ascorbic acid (86.5%), succinic acid (78.8%), palmitic acid (69.3%), alanine (61.4%), leucine (45.7%), and GABA (30.5%). For Method 2, the average recovery percentage was 35%, with the highest recovery observed for palmitic acid (181%), followed by succinic acid (85.6%), citric acid (82.6%), and phenylalanine (0.4%). Method 4 achieved an average recovery percentage of 28.3%, with citric acid showing the highest recovery (130.8%), followed by succinic acid (79.1%), palmitic acid (67.2%), and phenylalanine (6.2%). In the case of Method 1, the average percentage recovery was 22.2%, with succinic acid demonstrating the highest recovery (82.3%), followed by citric acid (72.3%) and palmitic acid (67.6%). Method 3 exhibited the lowest average percentage recovery at 20.8%, with succinic acid achieving the best recovery (80.1%), followed by citric acid (65.6%), palmitic acid (50.7%), and phenylalanine (27%). It is noteworthy that some compounds showed recoveries above 100%. This can be attributed to the fact that the QC samples used during analysis were compiled from human urine rather than synthetic urine, ensuring a realistic representation in terms of robustness during this investigation. Thus, many of these compounds may already be present in the samples.

Alanine, GABA, ascorbic acid, and leucine exhibited poor extraction efficacy across all methods, resulting in a 0% recovery, except for Method 5 (DA), which extracted all these metabolites. It is well-known that the polarity of OAs can significantly influence their extraction efficiency. Acidic compounds containing more hydroxyl groups tend to have lower recovery efficacy, as observed with ascorbic acid, which is very acidic with four hydroxyl groups. Mouskeftara et al. (2021)^26^ also reported significantly lower GABA signals when using ethyl acetate for analyte extraction compared to an acidic extraction with methyl tert-butyl ether (MTBE) or a more commonly used approach involving methanol (MeOH). Their study suggested that a combination of MeOH and methoxyamine with N-methyl-N-(trimethylsilyl)-trifluoroacetamide (MSTFA) 1% TMCS derivatization generally enhanced efficiency. However, BSTFA 1% TMCS exhibited a preference for certain acids, while MSTFA 1% TMCS yielded lower peaks for succinic acid. In contrast, our study achieved a recovery efficiency of 79% or more across all the OA extraction methods using BSTFA 1% TMCS. For future studies employing an LLE approach, exploring the potential use of MTBE in combination with acetonitrile is worth considering. Additionally, Pasikanti et al. (2008)^27^ compared various derivatization agents [BSTFA, MSTFA, and N-methyl-bis(trifluoroacetamide) (MBTFA)] used in urinary GC–MS metabolomics. In terms of the number of observed peaks, peak intensity, and reproducibility, both BSTFA and MSTFA demonstrated equal derivatization efficiency. Since MBTFA is more effective in derivatizing secondary and tertiary amine groups, they also explored the combination use of MBTFA with BSTFA. When MBTFA derivatization was preceded by BSTFA derivatization, more amino acids were detected, however, the integrated peak areas had poor reproducibility. Based on these findings, the authors recommended a preference for BSTFA as the derivatization agent.

Tryptophan and tyrosine had 0% recovery across all extraction methods, however, this was expected since GC systems are not ideal for detecting amino acids with great precision^25,28^. By comparing the various OA extraction techniques, Methods 3 and 4 were able to recover the most amounts of phenylalanine. This is most likely due to the addition of an oximation step, which produces methoxime derivatives that are more stable and improves peak identification^26,29,30^. The metabolite recovery ranking, however, shows that acetonitrile is required for higher extraction efficacy^22^ and that oximation alone is insufficient to recover broad ranges of metabolite classes.

However, it is important to exercise caution when interpreting metabolomics results, with particular attention to the specific methodology employed for sample extraction, as well as the analytical platform used for analysis. In the field of metabolomics, the choice of analytical platform is a critical consideration when designing a study. Each platform possesses its unique strengths and limitations in terms of detecting specific metabolites or metabolite classes. For instance, nuclear magnetic resonance (NMR) is particularly adept at detecting sugars, while liquid chromatography (LC) platforms excel in the analysis of amino acids, which gas chromatography (GC) systems may have limitations in detecting with high precision. However, GC platforms offer high efficiency, sensitivity, and reproducibility, making them well-suited for conducting non-targeted or so-called "discovery mode" metabolomics research where a broad range of metabolites needs to be analysed. In the context of this study, our focus was primarily on the application of methods optimised for a GC–MS platform, which has its own unique advantages. Researchers should carefully select the analytical platform that aligns with the specific metabolites of interest and the objectives of their metabolomics study to ensure meaningful and accurate results. While our methods may not be universally applicable, they provide valuable insights into the efficient extraction and analysis of metabolites using a GC–MS platform, and future studies exploring other platforms for specific metabolites or metabolite classes are certainly warranted.

### Selecting the most appropriate method for non-targeted GC–MS application

After each validation parameter was investigated, each method was ranked based on its overall performance, and a cumulative score was calculated for each method (see Table 3). Since a top ranking would result in a lower cumulative score, the method with the lowest cumulative score should theoretically be the superior method.

**Table 3:** Method rankings based on validation parameters, and an overall cumulative score of each.

	Repeatability	Metabolome coverage	Metabolite recovery	Cumulative score
Method 1 (original in-house)	4	5	4	13
Method 2 (acetonitrile addition)	5	2	2	9
Method 3 (two-step derivatization)	2	4	5	11
Method 4 (combination of Methods 2 & 3)	3	3	3	9
Method 5 (direct analysis)	1	1	1	3

The ranking provided here can be considered for future studies related to semi-targeted metabolomics^31^. It should be noted that repeatability is subjective to the analyst’s ability. Naturally, the fewer analytical steps involved, the simpler the method would be, and hence perform better in terms of repeatability. When an investigation requires a semi-targeted approach to extract OAs, for example, Methods 2 or 4 should be considered for optimal results. Furthermore, both options allowed for a high number of unique compounds and the overall recovery of organic and fatty acids was good for both methods. On the other hand, a non-targeted strategy examines the complete metabolic state^32^ and would therefore potentially benefit more from taking the percentage of undetected compounds into account, as the investigation would need to identify multiple groups of substances or different compound classes simultaneously^33^.

Considering the aim of the investigation, i.e., to optimise a low-volume urine preparation procedure for non-targeted GC–MS analyses covering various compound classes, Method 5 outperformed all other methods, consistently ranking first in every validation parameter. Therefore, we attempted to further optimise Method 5 by determining if an additional drying step between two-step derivatization (Method 6), and urease sample pre-treatment, also considering the effect of heating versus sonication (Method 7), is beneficial.

### Additional drying between two-step derivatization

**Figure 3:**
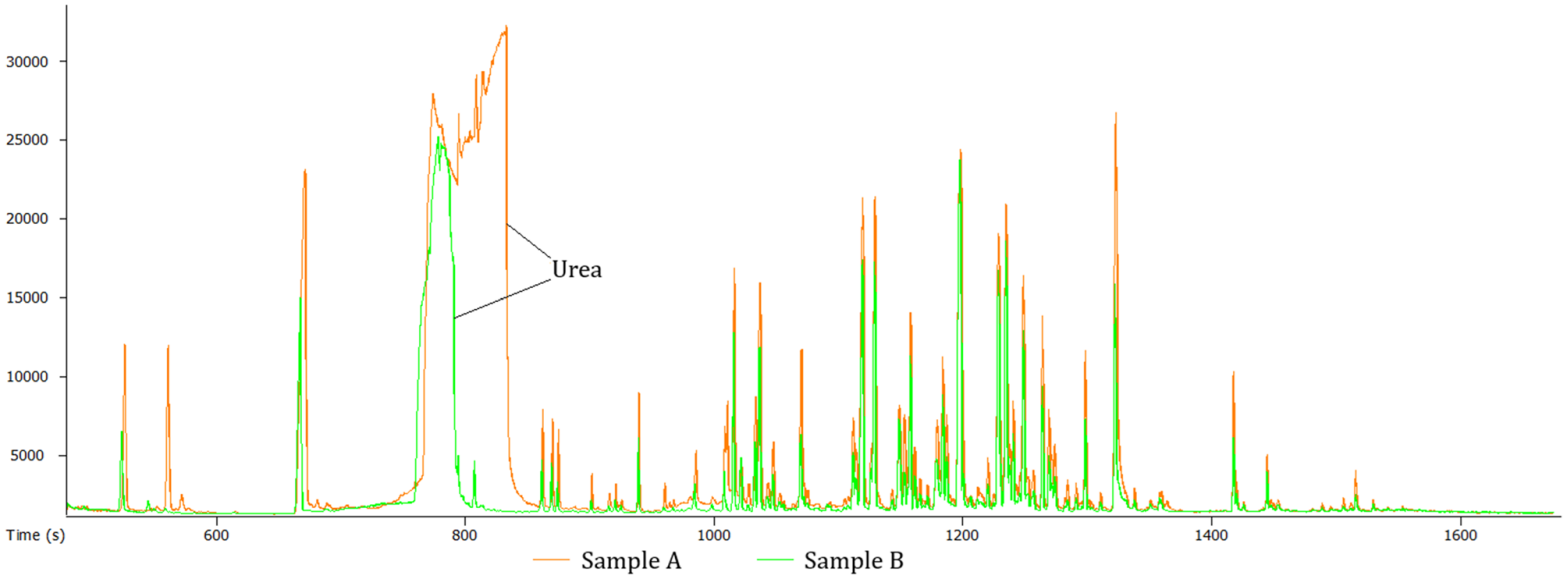
The total ion chromatograms (TICs) showing the effect of an additional drying step. Sample A (orange) was subjected to two-step derivatization while Sample B (green) was subjected to an additional drying step between two-step derivatization. Sample B showed a significant reduction in the signal of urea, which is considered advantageous.

For this part of the investigation, samples were extracted as described in Section "Method 6 (additional drying step)". When considering the total ion chromatograms (Fig. 3) from Sample A (orange; traditional two-step derivatization) and Sample B (green; additional drying between two-step derivatization), the additional drying step seems to reduce the signal of urea significantly, which is considered advantageous.

Although the average peak height was visually higher in the conventional two-step derivatization samples, certain metabolite classes still displayed higher reported areas. One such group was amino acids, which has also been reported by Liebeke and Puskás (2019)^15^ to be higher following an additional drying step samples. Moreover, the inclusion of an additional drying step results in a reduction of signal for specific metabolites often referred to as “junk compounds”, which commonly arise from reagent artifacts.

### Urease sample pre-treatment

To evaluate the potential advantages of urease sample pre-treatment and examine the impact of heating versus sonication, sample analysis was conducted following the procedure outlined in Section "Method 7 (urease pre-treatment)". The five previously mentioned sample groups (UT-H, NT-H, WT-H, UT-S, and NT-S) were compared in two separate experimental sets. The previously described criteria of repeatability, metabolome coverage, and metabolite recovery (Sections "Repeatability", "Metabolome coverage" and "Metabolite recovery") were employed to assess the outcomes of both experiments. Firstly, the effect of urease pre-treatment (during which samples were subjected to heating only, i.e., UT-H, NT-H, and WT-H) was evaluated. The results obtained in every validation parameter is indicated in Table 4. Here, the water-treated samples had the lowest cumulative ranking score between the three groups, and hence performed the best, with the urease-treated samples having the most unfavourable ranking in each assessment.

**Table 4:** Methods rankings based on validation parameters, when comparing urease-treated, urease-non-treated, and water-treated samples.

	Repeatability	Metabolome coverage	Metabolite recovery	Cumulative score
Urease-treated samples (UT-H)	3	3	3	9
Urease-non-treated samples (NT-H)	2	2	2	6
Water-treated samples (WT-H)	1	1	1	3

Urease treatment effectively reduces the urea content in urine samples, but it can have additional effects beyond urea reduction, which can negatively impact sample performance. These effects are influenced by various factors. Firstly, undesired enzymatic reactions may occur since enzymes can catalyse reactions beyond the intended target, leading to chemical changes in the sample. These changes may include alterations in the metabolite profiles, degradation of certain compounds, or the generation of new metabolites as by-products of the enzymatic reactions. Such effects can impact the composition, stability, and integrity of the metabolites of interest, introducing variability and decreasing the accuracy of the analysis^20^.

Secondly, urease treatment may decrease the recovery of metabolites of interest due to chemical transformations or interactions with the enzymatic products generated during urea hydrolysis. If certain metabolites are poorly recovered or lost entirely due to urease treatment, it can result in gaps or biases in the metabolome coverage, compromising the accuracy and reliability of the analysis. Consequently, a comprehensive understanding of the metabolic profile may be hindered, potentially leading to incomplete or misleading interpretations of the results^7,16^.

Thirdly, water-treated samples may exhibit a slight dilution effect, enhancing the extraction capability and improving the detection and measurement of metabolites^16^. Dilution helps mitigate potential matrix effects or sample interferences that could affect the analysis. Matrix effects can occur when the components of the sample, such as salts or proteins, interfere with the detection and measurement of analytes. By diluting the sample, the concentrations of these interfering components are reduced, potentially minimizing their impact on the analysis^34^. Consequently, a more accurate and reliable measurement of the target analytes can be achieved. However, the optimal concentration range depends on the specific technique and analyte characteristics, as extremely high or low concentrations may present challenges in terms of sensitivity, linearity, or dynamic range.

Fourthly, urease activity itself can cause interference or unwanted changes in the sample due to its inherent catalytic activity^35,36^. This interference can manifest as chemical interactions or alterations in the stability, integrity, or concentration of metabolites of interest.

Lastly, urease treatment adds complexity and additional steps to the sample preparation process, increasing the risk of errors or variability. Each additional step presents an opportunity for introducing artifacts, contamination, or analyte loss^16^. Inaccurate or inconsistent handling, mixing, or transferring of samples during urease pre-treatment can impact the accuracy, precision, and reproducibility of the analysis.

**Figure 4:**
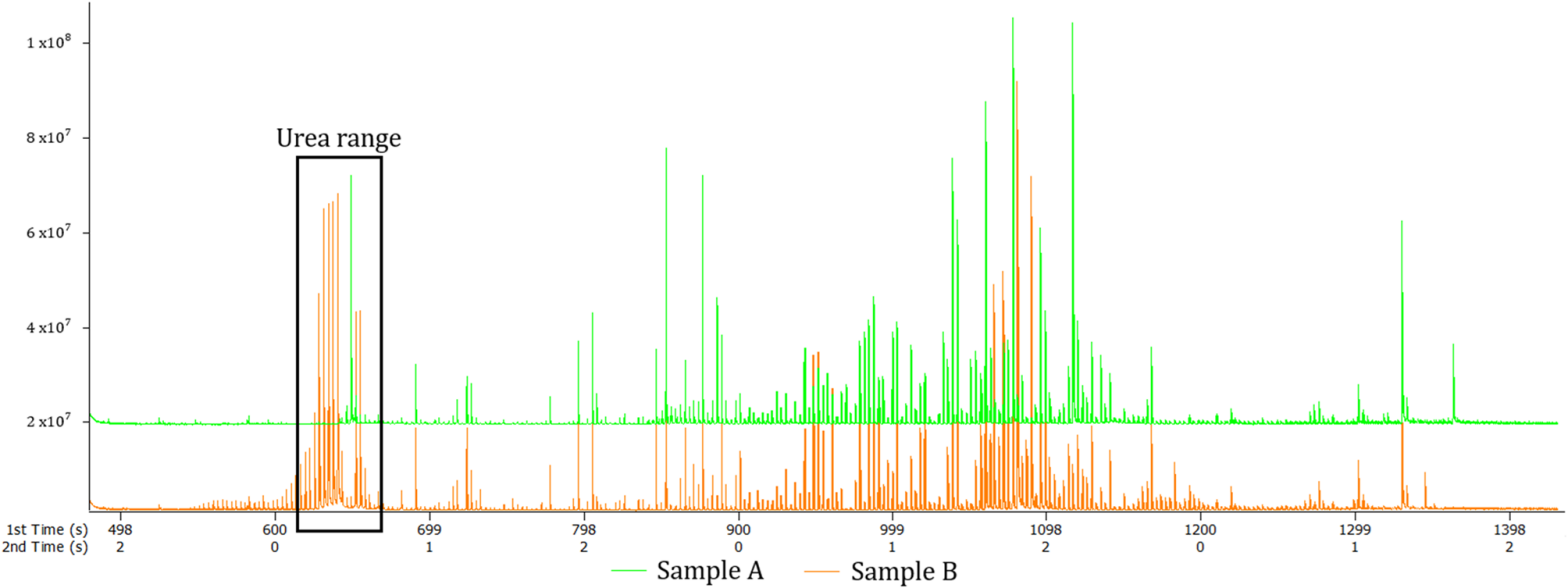
The total ion chromatograms showing the effect of urease sample pre-treatment. Sample A (green) was subjected to urease pre-treatment while Sample B (orange) shows a urease-non-treated sample.

The total ion chromatograms (Fig. 4) of a urease-treated sample (Sample A; green) and a urease-non-treated sample (Sample B; orange) were compared. Based on visual inspection, urease pre-treatment indeed reduced the amount of urea significantly. However, urease also reduced the overall metabolic signals, likely due to the various factors discussed above. This again highlights the detrimental effect that urease pre-treatment can have when non-targeted metabolomics studies are performed. In disease characterisation studies, for example, the metabolic profiles of patients need to remain unchanged, hence urease pre-treatment would not be recommended for such patient samples.

Hereafter, an additional experiment was conducted to examine the impact of heating versus sonication during urease pre-treatment to determine if this could increase the benefits of such an approach. Thus, UT-H, NT-H, UT-S, and NT-S were compared (Section "Method 7 (urease pre-treatment)") and again assessed based on the previously described criteria of repeatability, metabolome coverage, and metabolite recovery (Sections "Repeatability", "Metabolome coverage" and "Metabolite recovery"), results of which are indicated in Table 5.

**Table 5:** Method rankings based on validation parameters, when comparing heating and sonication following urease pre-treatment.

	Repeatability	Metabolome coverage	Metabolite recovery	Cumulative score
Urease-treated: Heating (UT-H)	4	4	4	12
Urease-treated: Sonicate (UT-S)	2	3	2	7
Urease-non-treated: Heating (NT-H)	1	2	3	6
Urease-non-treated: Sonicate (NT-S)	3	1	1	5

Based on the results, an approach of urease non-treatment, regardless of heating or sonication, consistently outperforms approaches where samples are subjected to urease pre-treatment, demonstrating superior performance, and yielding more reliable results. These findings align with the conclusions reached by Kim et al. (2020)^20^, who observed that urease pre-treatment may not be as advantageous as initially believed. Their study revealed that urease pre-treatment introduced artefacts into the metabolite profiles, potentially leading to misinterpretation of results. Other studies, however, observed that urease pre-treatment samples exhibited higher concentrations compared to urease non-treated^18^ and water-treated samples^16^. Nonetheless, Palmas et al. (2018)^18^ observed a small number of metabolites in urease non-treated samples that exhibited higher concentrations compared to the sonicated urease-treated samples, aligning with our findings. Although the exact origin remains unknown, they postulated that some of the ammonia generated during the urea conversion process in sonication may influence the concentrations of various metabolites. When urea undergoes conversion into ammonia and carbon dioxide, the former may react with acidic species, resulting in the formation of ammonium salts. Since GC–MS cannot detect salts, it may explain the reduced metabolite levels. Furthermore, this study noted that a longer sonication duration (1-h) combined with 200 μL urease solution is optimal, suggesting a potential avenue for future urease pre-treatment studies involving sonication.

Considering the continued contradictory results regarding urease pre-treatment, researchers should carefully assess its effects and evaluate its potential benefits primarily for specific analyses focused on urea-related measurements, while also ensuring the reliability and validity of the results. It is important to evaluate the trade-off between urea reduction and the potential compromise in metabolite recovery to ensure the most accurate and comprehensive analysis of the metabolome. If urease pre-treatment is deemed necessary, sonication offers numerous advantages in terms of efficiency, homogeneity, accessibility, avoidance of thermal degradation, and versatility, making it a preferred option to heating.

In terms of heating versus sonication when performing urease pre-treatment, results indicate that sonication is the preferred method to catalyse this reaction. Sonication can be more advantageous than heating due to several reasons, including (i) efficient and rapid mixing and agitation of the sample; (ii) achieving a more homogeneous distribution of the urease enzyme throughout the sample; (iii) facilitating the penetration of the enzyme into cellular compartments or structures, which may be more difficult to access via heating alone; and (iv) avoidance of thermal degradation of sensitive compounds present in the sample as it does not involve significant temperature increases^37,38^.

### Implications of the findings

The implications of our findings are significant for clinical, nutritional, and biological investigations focused on examining metabolic abnormalities associated with various pathophysiological conditions. Depending on the specific research objectives, our extraction methods can serve as valuable guidelines. Disease characterisation, for example, typically employs non-targeted metabolomics approaches (such as Method 6) to obtain comprehensive metabolic information about individuals^39^. The data analysis can lead to the development of new hypotheses, facilitating the exploration of previously undiscovered disease mechanisms, host–pathogen interactions and adaptations^21^. When disease characterisation indicates abnormalities in OA or urea profiles, researchers may opt for a more semi-targeted approach (such as Methods 2 or 4, as per our results) or targeted approach (Method 7 with sonication), respectively.

While this study primarily adopts a “discovery mode” process with non-targeted methods, aimed at comparative analysis between different techniques to identify the superior method, it is important to acknowledge the limitations of a non-targeted approach. A targeted metabolomics approach, while providing more accurate quantitative results, focuses only on a predetermined list of metabolites^40^. The optimised extraction method presented here holds promise for application in clinical laboratories, especially where time and resources are limited. This efficient method minimises extraction solvents, making it suitable for high-throughput analysis. Its potential to enhance treatment strategies, overcome diagnostic limitations, and enable prompt medical intervention in disease cases makes it a valuable tool for researchers seeking to optimise urinalysis and make informed decisions aligned with their research objectives.

## Conclusion

Since non-targeted GC–MS metabolomics aims to investigate multiple metabolite classes at various concentrations, optimal compound extraction is essential. Five urine sample preparation methods were evaluated in terms of repeatability, metabolome coverage, and metabolite recovery, with the aim of determining which is better for non-targeted GC–MS metabolomics applications. A low-volume DA method outperformed all other methods, consistently ranking first in every validation parameter. This method identified 91 unique metabolites, from multiple compound classes, and showed high metabolite recovery. An extraction method that can simultaneously identify all the metabolites in a urine sample is not yet available, mostly due to the complexities and the variety of physiochemical properties of metabolites. Nonetheless, this DA method requires little sample volume, have few analytical steps (which would be more time efficient, cost-effective, and repeatable), and can extract as much of the metabolome as possible, making it ideal for non-targeted urine GC–MS analysis.

Furthermore, the DA approach was optimised by incorporating an additional drying step between two-step derivatization but did not benefit from urease sample pre-treatment. Indeed, we recommend that urease pre-treatment should be reserved for specific analyses targeting urea-related measurements. If urease pre-treatment is necessary, sonication is recommended due to its numerous advantages, including efficiency, homogeneity, accessibility, avoidance of thermal degradation, and versatility compared to heating.

### Supplementary Information

Supplementary Information.

## Data Availability

All data is available on BioStudies (Accession Number S-BSST1138).
