# Supplementary material for: Optimising a urinary extraction method for non-targeted GC–MS metabolomics
Source: Sci Rep. 2023 Oct 16;13:17591. doi: 10.1038/s41598-023-44690-7 (PMC10579216; doi:10.1038/s41598-023-44690-7)
Supplement: Supplementary file 1 — Supplementary Information. [file 41598_2023_44690_MOESM1_ESM.docx]

**Supplementary information**

For GC‑MS analysis, the primary oven program is depicted in Figure S1. The initial temperature was 70°C, which was held for 1min. The temperature was then ramped up as follows: 5°C/min to 100°C, 10°C/min to 160°C, 13°C/min to 230°C, and finally 20°C/min to 300°C, which was held for 2 min. The secondary oven was programmed identical to that of the primary oven, except for a +5°C at each interval. The total run time of each sample was ≈24min.


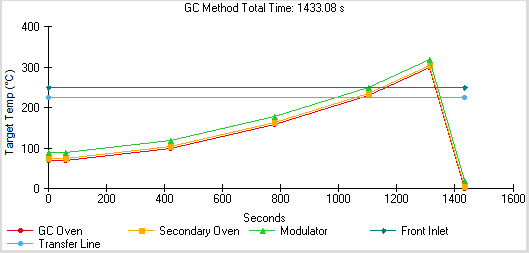


Figure S1: Temperature ramping during GC-MS analysis.
